# Improving retention and HIV viral load suppression among adolescents living with HIV in TASO Soroti and TASO Mbale centers of excellence using Operation Triple Zero model: a before and after study protocol

**DOI:** 10.1186/s43058-023-00449-9

**Published:** 2023-06-12

**Authors:** Bonniface Oryokot, Andrew Kazibwe, David Kagimu, Abraham Ignatius Oluka, Darlius Kato, Yunus Miya, Michael Bernard Etukoit, Eleanor Namusoke-Magongo

**Affiliations:** 1 The AIDS Support Organization (TASO) Uganda, Kampala, Uganda; 2 University of Suffolk, Ipswich, UK; 3 Makerere University College of Health Sciences, Kampala, Uganda; 4 AIDS Control Program, Ministry of Health, Kampala, Uganda

**Keywords:** HIV, Adolescents, OTZ, Viral load, Retention

## Abstract

**Background:**

Retention in care and HIV viral load suppression remains sub-optimal among HIV positive adolescents in many settings including TASO Uganda, despite the implementation of interventions such as regimen optimization and community-based approaches like multi-month drug dispensing. To this end, the implementation of additional intervention is urgently required to address gaps in current programming which include inadequate centralization of the HIV positive adolescents and their caregivers in the designs. This study, thus, proposes to adapt and implement the Operation Triple Zero (OTZ) model in TASO Soroti and Mbale centers to improve both retention and viral load suppression among the adolescents living with HIV.

**Methodology:**

A before and after study design is preferred, employing both qualitative and quantitative approaches. To identify barriers and facilitators to retention and HIV viral load suppression among the HIV positive adolescents, secondary data, focused group discussions, and key informant interviews will be used to understand perspectives of the adolescents, their caregivers, and the health-workers. The Consolidated Framework for Implementation Research (CFIR) will help in designing the intervention, while Knowledge to Action (K2A) will support the adaptation process. To test the intervention, Reach, Effectiveness, Adaption, Implementation and Maintenance (RE-AIM) framework will be used. A paired *t*-test will be used to compare means of retention and viral load suppression in the before and after study periods.

**Discussion:**

This study aims at adapting and implementing the OTZ model in TASO Soroti and Mbale Centers of Excellence (COEs) to attain optimal retention and HIV viral load suppression rates among the HIV positive adolescents in care. Uganda is yet to adapt the touted OTZ model and findings from this study will be important in providing the necessary lessons to inform a policy shift for potential scale up of the model. Furthermore, results of this study could provide additional evidence for the effectiveness of OTZ in attaining optimal HIV treatment outcomes among the adolescents living with HIV.

Contributions to literature

- Findings will likely provide additional implementation strategies to help strengthen adaptation of the OTZ model in other settings. Lessons learnt in the two settings will be vital for scaling up the model in the country should the ministry of health decide to do so.
- Additionally, findings may also strengthen the merits to the effectiveness of OTZ in improving treatment outcomes among the adolescents living with HIV.
- The study employs a mixed approach, placing the adolescent and caregiver at the center of the intervention enabling their perspectives to contribute to the intervention design. Thus, the findings could enrich our understanding of optimally managing HIV positive adolescents using differentiated service delivery approach.

## Background

The universal coverage of antiretroviral therapy (ART) has increased access to HIV treatment among the HIV positive children and adolescents globally. This scale up is expected to reduce morbidity and mortality among the children and adolescents aged below 20 years living with HIV (CALHIV), contributing to ending the AIDS epidemic. Like many countries in the region, Uganda adopted the “test-and-treat” policy among children and adolescents in 2013 to accelerate attainment of epidemic control [1]. However, for this to happen, the CALHIV who initiate ART need to remain in care and achieve sustained HIV viral load suppression [2, 3].

Unfortunately, these treatment outcomes have remained sub-optimal among the CALHIV [2]. Recent evidence indicates that mortality among the adolescents has increased by 50% [4], yet the same decreased significantly among the general population. Furthermore, available evidence indicates that retention remains much lower among the adolescents living with HIV than the rest of the population living with HIV (PLHIV) [5]. For example, a Ugandan study established that 1-year retention among the adolescents living with HIV was 29% in southwestern Uganda [6]. A more recent study by Muwanguzi et al. [7] reported a slightly better finding, with 65% of adolescents and young adults aged 15–24 years old in southwestern Uganda retained in care within 1 year. Indeed, routine programmatic data from Uganda showed that 1-year retention rate among the children aged below 10 years was 87%, while it was 56% for adolescents aged 10–19, far below the expected 95%. In terms of HIV viral load (VL), the CALHIV still remain disproportionately affected. In Uganda, a 2017 HIV population impact assessment found a VL suppression rate of 49% among the same population [8]. Similarly, by the end of June 2021, routine programmatic data from Ministry of Health (MOH) indicated that overall, 41% of CALHIV were virally suppressed at population level.

A plethora of socioeconomic and cultural factors has been documented as responsible for these poor HIV treatment outcomes among the CALHIV. These factors include poor family support, poor adherence, stigma, food scarcity, non-disclosure of HIV status, unfriendly school or health service environments, peer factor, and young age [4, 9–12]. Indeed, unreliable supply chain, rude health workers, lack of transport means, and limited time offered to adolescents were found to negatively affect retention in selected clinics in South Africa [13]. Furthermore, a study in Cambodia found that older adolescents aged 17 years or more, longer duration on ART, and receiving care from adult clinics were associated with non-suppressed viral load [14]. In addition, a Kenyan study reported that caregiver non-suppressed VL status and young age were associated with non-suppression among children [15]. Finally, other critical factors include co-infection with tuberculosis (TB), mental illness, erratic transitions of CALHIV through the different care clinics, and non-patient centered service provision [5, 16–18]. Despite acknowledging these well documented factors, many HIV programs, including The AIDS Support Organization (TASO) Uganda, continue to struggle to achieve optimal treatment outcomes. For example, TASO Soroti and Mbale Centers of Excellence (COE) had VLS of 80% and 86% respectively by the end of June 2022. Noteworthy, TASO has implemented the Youth and Adolescent Peer Supporter (YAPS) model since March 2021, transitioned the adolescents to optimal regimens such as dolutegravir (DTG) and enhanced community-based approaches such as multi-month dispensing [19]. Yet, HIV treatment outcomes in this sub-population remain sub-optimal. Perhaps, these interventions mainly focus on the client, albeit with their limited input in actual program designs. To this end, we believe that implementing a more comprehensive model such as Operation Triple Zero (OTZ) could improve HIV treatment outcomes including retention and VLS among the ALHIV. OTZ model was first successfully implemented in Kenya, subsequently adapted in Zambia, Ethiopia, and Lesotho with good outcomes. It aims at achieving zero missed appointment, zero missed pill, and zero VL through empowering the health worker, the caregiver, and the adolescents themselves. This study, therefore, aims to adapt, implement, and evaluate OTZ in the TASO setting in a bid to improve retention and VLS among the adolescents.

## Methodology

### Study design

We shall use a before and after study design, with a mixed method approach of data collection. Both quantitative and qualitative data will be collected in parallel.

### Study objectives

There are three specific study objectives and include:

- To identify barriers and facilitators to retention and VLS among the adolescents living with HIV in TASO Mbale and Soroti COEs
- To improve retention and VLS among ALHIV by implementing the OTZ model in TASO Soroti and Mbale COEs
- To test the effect of OTZ on retention and VLS among ALHIV who receive care at TASO Soroti and Mbale COEs by the end of December 2023

### Study population

The study population is as follows: ALHIV who are virally non-suppressed. The 2020 Uganda national HIV care and treatment guidelines define VL non-suppression as having HIV VL copies above 1000/mL [19]. However, this definition could change in the future, and the study will adopt accordingly as and when the ministry of health guideline changes. All PLHIV with non-suppressed VL are expected to access intensive adherence counseling for at least three consecutive months till adherence becomes optimal, then re-monitored for VLS [19]. Those whose repeat VL remain non-suppressed, despite optimal adherence, are then subjected to drug resistance tests for further assessment [19]. Intensive adherence counseling, a targeted and effective intervention, is expected to cause a re-suppression rate of at least 70% [20].

Despite the adoption of intensive adherence counseling (IAC) since 2016, the outcomes have been sub-optimal. For example, a study by Nasuuna and colleagues reported a re-suppression rate of 23% [21] among the CALHIV. In a more recent study from Ugandan military facilities involving all virally non-suppressed PLHIV, 48.2% of the individuals achieved re-suppression [22]. The effectiveness of IAC has remained suboptimal probably due to other factors such as treatment illiteracy among ALHIV and their caregivers, inadequate fidelity to guidelines by health workers, and inadequate involvement of peers. We expect to avert these shortcomings by implementing the OTZ model to enhance uptake of IAC. Other study participants will include caregivers of the ALHIV, health care workers, and peers.

### Study setting

TASO is the largest indigenous not-for-profit organization in Uganda that provides comprehensive HIV services. It has 11 centers of excellence (COEs) spread across the country providing care and treatment services to nearly 80,000 PLHIV including approximately 4000 CALHIV. The study shall be conducted in two TASO COEs of Soroti and Mbale, which currently have the lowest VL suppression rates among the CALHIV. TASO Mbale and TASO Soroti have 58 and 47 virally non-suppressed ALHIV, respectively, with VLS rates of 86% and 84%, respectively. Overall, TASO Soroti and TASO Mbale have approximately 300 and 420 ALHIV in care, respectively. Each of the centers runs a separate model of pediatric and adolescent HIV clinics, supported by peers through a national program known as YAPS. YAPS generally have reasonable levels of education and can read and write in English. In addition, they have undergone some basic training in peer adherence counseling and are expected to provide ongoing counseling to other CALHIV.

### Sample size and sampling procedure

#### Quantitative component

TASO Mbale has approximately 420 ALHIV in care, and TASO Soroti has 300. We shall use a census method to collect data on ALHIV aged 10-17 years who were active in care at the two TASO centers in the April-June 2022 quarter. This will enable us determine one-year retention rate and also maintain all those enrolled in the program throughout the project period before transitioning to adult care.

#### Qualitative component

We will use purposive sampling approach to recruit respondents. Both focused group discussions (FGD) and key informant interviews (KII) will be conducted. Details of this are expounded under the “Data collection” section. Noteworthy, we shall conduct 8 FGDs in the two COEs, involving groups of 5–6 individuals. Four groups shall include all non-suppressed ALHIV and caregivers of non-suppressors, while the remaining groups shall include caregivers of suppressed ALHIV and adolescents with suppressed VL. KII shall be done on key health workers—two counselors, two clinicians, two heads of department, two caregivers, four ALHIV (two non-suppressed and two with suppressed VL), and two peers. Overall, the engagements will be face-to-face and at the clinic premises.

*Inclusion criteria*

- All ALHIV who are aged 10-17 years of age and active in care in the two COEs during the April-June 2022 quarter.
- Caregivers of the ALHIV
- Health workers directly involved with ALHIV in the two facilities

*Exclusion criteria*

- Individuals who do not speak either English, Luganda, Ateso, or Lugisu languages
- Individuals who will join the COEs after the study has begun and those aged 18 years or older.

### Study approach

#### To identify barriers and facilitators to retention and viral load suppression among the ALHIV in TASO Mbale and Soroti COEs

This objective seeks to appreciate current HIV care programming in the two COEs and identify potential bottlenecks and promoters from the perspectives of caregivers, ALHIV, and health workers. This is aimed at understanding contextual issues so that effective strategies may be designed to enhance chances of a successful OTZ implementation. We will use both quantitative and qualitative methods to address this objective.

We propose to use the Consolidated Framework of Implementation Research (CFIR) to identify barriers and facilitators of optimal retention and viral load suppression among the ALHIV in the TASO Uganda setting. CFIR is a widely used framework of implementation science in this regard and has five domains and 39 constructs for adaptation [23, 24]. Two domains (inner and outer setting) of CFIR shall be used to identifying the facilitators and barriers that currently exist. Both quantitative and qualitative approaches shall be used to identify the barriers and facilitators of the current pediatric HIV programming. Quantitative data shall be collected using a questionnaire while a semi-structured interview approach shall provide qualitative data. In addition, 4 focused group discussions per facility shall be conducted among the caregivers and the ALHIV who are virally non-suppressed to identify some of the key obstacles to effective ART utilization. Furthermore, 2 FGDs shall be conducted among caregivers of adolescents with suppressed VL and ALHIV with suppressed VL to appreciate their recipes for success. Finally, current adolescent HIV programming in the two settings will be explored to enable effective design of appropriate OTZ implementation.

**Table:** 

Outer and inner settings
Semi-structured interview questions will be used to elicit views of key stakeholders as detailed below: • Health unit teams (medical services technical lead, counselor, center program manager, peers, clinician, psychosocial and community linkage officer, and monitoring and evaluation officer). The constructs of leadership engagement, availability of resources, and tension for change will be used to guide data collection • *Caregivers*. These are individuals such as biological parents, teachers, guardians, and siblings who provide routine assistance to the ALHIV. The kind of assistance may include accompanying them to the health unit, reminding them to swallow ART, education, and other socioeconomic services • *The adolescents living with HIV*. An adolescent is defined in this study as an HIV positive individual who is aged 10–19 years old The construct of client needs and resources will be used to guide data collection from caregivers and adolescents

#### To improve retention and VLS among ALHIV by implementing the OTZ model in TASO Soroti and Mbale COEs

This objective seeks to build on the findings on the barriers and facilitators to retention and VLS by designing effective, context-specific strategies to implement OTZ in the two TASO COEs. This is aimed at improving VLS and retention through adapting OTZ in the most context-friendly manner.

OTZ aims at attaining zero missed appointment, zero missed pill, and zero VL among the ALHIV. The model also promotes zero stigma, zero death, zero sex for abstaining adolescents, and zero mother-to-child transmission of HIV for those who become pregnant. It is an asset-based model that encourages active participation of adolescents and young people by enjoying more positive health behaviors [25]. As a Kenyan initiative that began in 2016, OTZ heavily relies on peers, also known as OTZ champions, to provide treatment literacy that empowers young HIV positive people to be more responsible for their own health [26, 27]. The model has comprehensive packages for health workers, caregivers, and the adolescents or young people living with HIV [25]. The package for health workers entails training on clinical assessment and HIV treatment, communication with adolescents, nutrition, mental health, sexual and reproductive health, and transition to adult clinics. The aim is to instill competence among the health workers to effectively manage and empower the ALHIV. For caregivers are basic information on HIV/AIDS, disclosure, adherence, nutrition, and discrimination. Lastly, adolescents received messages such as the importance of joining the OTZ club, leadership, adherence, transition to adult clinics, celebrating those who achieve VL suppression, and general health literacy. This is aimed at empowering the clients to make positive life/health choices for a more sustained life.

The model enhanced VL suppression rates in Kenya among the adolescents from 71 to 82% within 6 months of implementation, and self-reported adherence increased from 88% to at least 96% between 2016 and 2019 [25, 26]. Furthermore, Kenya has scaled up the model from 70 beneficiaries in one health unit to more than 29,795 in 27 different counties with high HIV burden [25]. The OTZ initiative was adapted in Zambia to enhance health outcomes among the adolescents and young mothers, including zero viral load, zero mother-to-child transmission, and zero teen pregnancies [28]. Other countries implementing OTZ adaptations include Nigeria and Ethiopia. In general terms, the use of peers has been widely documented as successful in different settings, including the Zvandiri interventions of Zimbabwe and others in south Africa [17, 29]. Indeed, a similar program was previously implemented in TASO Mbale clinic and viral load improved among the adolescents from 61 to 97% [30]. Importantly, OTZ is now among the models recommended by the WHO for improving health outcomes among the adolescents and young people living with HIV.

Despite the success of OTZ model in Kenya, it has not been adapted in Uganda. Well, the country is currently implementing a youth-led initiative known as Youth and Adolescent Peer Supporter (YAPS), which also relies on the assistance of HIV positive adolescents and young people to support their colleagues. However, it is less robust, with limited engagements of caregivers and health workers. Since its inception in early 2021, the effect remains minimal in many settings including within TASO. Indeed, viral load suppression among the ALHIV remains below 85% in Uganda, as indicated by routine program data at national level. In TASO, there are two centers that continue to experience the worst VL suppression rates: Soroti at 80% and Mbale at 86% as at the end of September 2022. We, therefore, propose to implement the OTZ model at the two TASO COEs to enhance VL suppression rate. TASO Uganda is a not-for profit and largest indigenous organization providing comprehensive HIV services. It was founded in 1987 with a vision of ‘a world without HIV/AIDS.’ There are 11 service centers spread across the country including Soroti, Mbale, Tororo, Jinja, Mulago, Entebbe, Masaka, Mbarara, Rukungiri, Masindi, and Gulu [31]. Like elsewhere in Uganda, TASO centers are struggling to achieve VL suppression among the ALHIV.

The adaptation process:

We shall apply the knowledge-to-action (K2A) framework [32, 33] to adapt and design the OTZ intervention. The following steps will be taken:

1. We will engage the Kenyan team to further our understanding of the OTZ model. This will be done virtually, using zoom facilities that are readily available within TASO.
2. Furthermore, we shall engage the facility teams to identify and agree on the current most important challenge in the pediatric and adolescent HIV clinic that requires urgent attention.
3. Next, we shall appraise the OTZ intervention, looking at its validity and relevance to the TASO setting. We will introduce the OTZ concept to facility teams to facilitate adaptation.
4. This shall be followed by the adaptation stage. Activities will include facility teams weighing the value, usefulness, and the appropriateness of OTZ to their specific setting. This is expected to be a critical step, culminating in embracing the model or otherwise.
5. Furthermore, potential barriers to the implementation of OTZ within the TASO setting shall be discussed and mitigation strategies proposed.
6. Finally, we will design context-focused interventions to enhance opportunities of a successful implementation of OTZ in the TASO setting. This will also facilitate fidelity measurement.

#### To test the effect of OTZ on retention and VLS among the ALHIV in TASO Soroti and Mbale COEs

This aims at evaluating the effect of the intervention on the outcomes of interest-retention, VLS and adherence. It is an important endeavor as it provides opportunities for learning lessons about what worked, what did not, and how things could be improved which are critical for scaling up of evidence-based interventions.

The implementation process:

- We will develop an orientation schedule and standard operating procedures to implement the OTZ model.
- Identify and train implementers. Particularly, we will work with the facility teams to identify suitable OTZ champions, leveraging the YAPS that are currently available. Similarly, the study will encourage facilities to identify a pediatric counselor and clinician to effectively implement the activities.
- Study implementation will take place. The health workers identified (clinicians, counselors, and peers) shall be trained and mentored using the standard MOH tools for managing ALHIV. The peers, too, will be trained using their appropriate package.
- We will provide all MOH standard tools that are relevant to the study. This will include appointment registers, missed appointment registers, viral load request books, non-suppressors’ registers, and other relevant documents.
- We will identify virally non-suppressed ALHIV aged 10–17 years old per site and enroll all into the study. They will receive the standard care as enshrined in the MOH national guidelines.
- All the non-suppressors will be considered as one cohort and provided with comprehensive care package. A new non-suppressors’ register will be used to enroll all of them. Health workers will ensure each of the receives expected care every month for at least three consecutive months, updated in both the HIV care cards, TASO counseling form and non-suppressors’ register.
- Peers shall be expected to provide additional adherence support, both virtually and physically, by contacting caregivers or adolescents on phone. This will be done twice weekly until considerable level of adherence is achieved. In addition, the peers shall each be responsible for at least twenty non-suppressed ALHIV and monthly meetings will be conducted to ensure the adolescents are adequately empowered.
- We will hold one workshop at the beginning of the study involving the peers, non-suppressed ALHIV, and their caregivers. During this workshop, the adolescents will be engaged in a group to empower them on positive living, while their caregivers shall be trained using the MOH treatment literacy toolkit to empower them in improving ART provision to the adolescents.
- Project monitoring and evaluation

#### Testing the intervention

- We propose to use the Reach, Effectiveness, Adoption, Implementation and Maintenance (RE-AIM) framework to test the intervention and implementation strategies [34]. RE-AIM is a tested framework for enhancing uptake of public health services. It enables monitoring and evaluation using both quantitative and qualitative approaches. Indeed, it has been extensively used and found to be efficient in clinical settings [35]. The framework asserts that the effect or impact of a proven public health intervention can be realized if the effective intervention reaches a reasonable proportion of the targeted population, adequately adopted by a willing organization, implemented with fidelity, and maintained over time [36]. A study in Tanzania used the RE-AIM framework to evaluate the integration of the methadone and antiretroviral therapy strategy [37]. RE-AIM enabled the identification of barriers and facilitators of the optimal uptake of the intervention. Similarly, RE-AIM was also applied in Mexico to identify barriers and facilitators of pre-exposure prophylaxis (PrEP) among the men who have sex with men and transgender women [38]. Lastly, in Brazil, RE-AIM helped a team of researchers to evaluate the integration of the continuum of care monitoring (CCM) intervention [39]. Therefore, RE-AIM shall be used in both before and after evaluation of the OTZ implementation.
- Both quantitative and qualitative approaches will be used to test the interventions. A questionnaire shall be used to collect quantitative data on reach (overall number of study beneficiaries), implementation fidelity, and clinical outcomes (retention and VL suppression). Both semi-structured interviews of key informants and focused group discussions shall be used to collect data on selected implementation outcomes adoption and fidelity. Fidelity is the adherence to intervention protocol, amount of the intervention implemented, and quality of its provision [40]. Fidelity shall be measured through self-reports.

## Data collection, management, and analysis

### Data collection

#### To identify the barriers and facilitators to retention and viral load suppression among the ALHIV in TASO Soroti and Mbale COEs

For quantitative data:

We will use a questionnaire to abstract secondary data to determine potential barriers and facilitators to retention and VLS. Data sources shall include Uganda electronic medical records (EMR), Central Public Health Laboratory (CPHL) VL dashboard, registers, patient level files, and TASO management information systems. Key independent variables shall include age, sex, TASO site, VL status and result, adherence level, ART status including start dates, regimens, and baseline characteristics. Data will be collected from all the ALHIV active in care, 12 months prior to study period.

Primary and secondary outcomes of interest shall be VLS, retention in care, and adherence levels. VLS, as already stated previously, shall be defined as VL copies of less than 1000/mL or as defined by the prevailing ministry of health guidelines. Additionally, VL of at least 1000 copies/mL will be recognized as non-suppressed. On the other hand, retention will be defined as the proportion of ALHIV who remain engaged in care after 12 months of project implementation determined by current ART status being within 28 days of the most recent appointment date. It will be categorized as active for individuals who are within their next scheduled appointment, dead for those that died, transfer-out for those with documented movement to other facilities, and lost for those whose disengagement spans beyond 28 days of most recent appointment, while missed appointment will be for those whose disengagement is less than 28 days of the most recent appointment. Retention data will look at all ALHIV who were active in care 12 months before the study period begins. Lastly, adherence shall be categorized by good if at least 95% of drugs swallowed, moderate/fair if 85–94% ARVs swallowed, and poor if less than 84% of drugs swallowed, as reported by the ALHIV or caregiver. We will use self-report or pill count to gather data on adherence.

For qualitative data, key informant interviews (KII) using semi-structured interview guides shall be used to collect views from facility management teams, health workers, and peers. In addition, 4 focused group discussions (FGDs) shall also be used to gather additional information from the adolescents and their caregivers. We shall conduct two FGDs consisting of groups of caregivers and clients to understand barriers and potential facilitators of retention and VL suppression. Furthermore, two additional FGDs shall be done (1 for care givers of ALHIV with suppressed VL and ALHIV with suppressed VL) to pick more information that could potentiate VLS in the non-suppressed group. Research assistants will be trained and used to conduct the interviews using the guides developed and pre-tested to optimize rigor. Further data collection will be done using field notes. Overall, eight FGDs will be conducted in the two TASO COEs. The KIIs and FGDs will be conducted at the clinics using face-to-face approach. Three trained and experienced research assistants will support data collection.

#### To improve retention and VLS among ALHIV by implementing the OTZ model in TASO Soroti and Mbale COEs

We shall use qualitative approach employing KII to understand the current pediatric and adolescent HIV programming in the two TASO COEs. KII using semi-structured interviews will be done to collect data on current the most important bottlenecks in regard to retention and VLS among the ALHIV who seek care in TASO. Facility heads (center programs managers), heads of department (psychosocial team leads and medical services technical leads), pediatric and adolescent HIV service focal persons, pediatric counselors, and peers will be engaged to provide relevant insights. In addition, data on OTZ knowledge among the respondents, its potential relevance to the TASO setting, and barriers to effective implementation shall be gathered. This will facilitate effective intervention design that’s tailored toward the TASO context.

#### To test the effect of OTZ on retention and VLS among the ALHIV in TASO Soroti and Mbale COEs

Both qualitative and quantitative approaches shall be used. A questionnaire shall be used to collect quantitative data to measure reach and effectiveness of the intervention.

Qualitative data shall be collected using semi-structured interview questions to appreciate perspectives of the facility teams on the fidelity of the implementation of the OTZ model. The center heads, heads of department, pediatric clinic teams, and peers shall be interviewed at the end of the study period.

### Data management

Study data from registers and Uganda electronic medical records (EMR) shall be extracted and stored in Microsoft Excel version 2019 in password-protected devices. In addition, the PI shall store a copy in a password protected external hard-drive for back-up. Only de-identified data shall be shared within the public domains.

### Data analysis

For objective 1: To identify the barriers and facilitators to retention and viral load suppression among the ALHIV in TASO Soroti and COEs.

- Quantitative data shall be downloaded into Microsoft Excel 2019 for preliminary analysis, exported for further analysis in STATA Corp version 15. Descriptive statistics shall be summarized as frequencies, percentages, and mean and standard deviations for categorical and continuous variables respectively. Results of descriptive analysis will be presented as tables.
- Pearson’s chi-square shall be used to determine associations among the various categorical variables. Those with significant scores (*p*-value < 0.05, 95% confidence interval) shall be considered for multivariate analysis. Multiple regression model will be used to determine factors associated with retention and Viral load suppression/non-suppression.
- Qualitative data analysis shall be done using thematic deductive approach, guided by CFIR. We plan to use three coders.

#### To improve retention and VLS among ALHIV by implementing the OTZ model in TASO Soroti and Mbale COEs

- Qualitative data analysis shall be done using deductive thematic approach and document reviews.

#### To implement and test the adapted OTZ model

- Quantitative data: Basic analysis in MS Excel 2019 version, exported to STATA Corp version 15 for complete analysis. Descriptive statistics shall be presented as frequencies and proportions. Descriptive and inferential statistics will be employed to provide insights of performance against set targets. Inferential statistics will be used to analyze the effectiveness of OTZ on VLS and retention. Retention will be measured as ART status at 12 months before study period and at 12 months after initiating implementation. Retention will be categorized as active if a client stays engaged in care, lost if an ALHIV misses appointment for more than 28 days from the most recent appointment, transfer-out if ALHIV moved to another facility with documentation, and dead if the adolescent died. Meanwhile, adherence will be measured as good if at least 95% of the expected pills are swallowed in the previous 3 months, fair if 85–94%, and poor if 84% and worse. We will use pill count or self-report to capture the data. A *t*-test will be used to compare mean scores both before and after project implementation. Retention will be measured as proportions (for example number of active in care at study point of interest divided by the original cohort), and the same cohorts will be tracked over the study period. As such, a paired *t*-test will be used to determine the significance of any potential difference between the average retention rates in the before and after intervention. Trends shall be used to determine significant changes over the study period using Pearson’s chi square trend test. Finally, modified Poisson regression model with standard errors will be fitted to complete data to measure rate ratios, at 95% confidence interval (CI). Poisson model will be used to separately analyze factors associated with retention and VLS, presented as adjusted and unadjusted ratios. Finally, associations at *p*-values less than 0.05 will be considered significant.
- Qualitative data: We will use the deductive approach of thematic analysis for qualitative data. Qualitative data shall be used to explain reasons for the different levels of performance and also perspectives of the different stakeholders. Audio-taped recordings will be transcribed verbatim and used to complement field notes. Transcriptions in English and observations will be analyzed using codes and themes. Qualitative data will be analyzed at group level for ALHIV and caregivers, using content and thematic analysis, to categorize behavioral or verbal data for summarization, tabulation, and classification. Qualitative findings will be triangulated with those of quantitative to inform the adaptation of OTZ to the TASO setting. We will use the ATLAS.ti software for analysis.

#### Study variables for quantitative component

Exposure variables include age, sex, DSDM, caregiver VL status, disclosure status, distance from home to health facility, multi-month dispensing, and caregiver type relationship.

### Study outcomes

#### Primary outcomes

- Viral load suppression—VLS will be defined as ALHIV having copies of less than 1000/mL.
- Retention: categorized into active (if the client is at least within 28 days of the most recent appointment), lost (ALHIV who disengaged from care for more than 28 days from the most recent appointment date), transfer out (a client who moves to another facility), and dead (ALHIV who dies during the course of the study).

#### Secondary outcomes

Adherence—The proportion of anti-retroviral drugs swallowed in a given period of time (measured as number of drugs swallowed/total number of drugs expected to be swallowed). This will be categorized as good if at least 95%, fair if 85–94%, and poor if 84% and below. We will use both pill count and self-report to measure adherence where pill count is not possible.

### Implementation outcomes

Fidelity: The extent to which OTZ will be implemented as planned. We will use qualitative measures to study fidelity, using observation, document review, and self-report by the health workers. A checklist (Table 1) will be used to aid fidelity assessment.

**Table 1:** Checklist

Component	Strategy	Data collection method	Status (Y/N)	Rating	
**Adherence**	Kenyan team engaged	Self-report			
	Caregiver workshop conducted	Self-report/document reviews			
	Peers and health workers provided with basic training	Self-report/document reviews			
	Peers assigned Non-suppressed ALHIV	Self-report			
	Monthly OTZ meetings conducted	Document review/self-report			
		Self-report			
**Dosage**	Number of times the OTZ club meetings were held	Self-report			
		Self-report			
**Quality of intervention delivery**	Project monitoring and evaluation done	Self-report			
	Feedback provided in a timely fashion	Self-report			
	Support supervision done	Observation/self-report			
	CQI projects done to support intervention delivery	Self-report/document review			
**Participant responsiveness**	Caregivers exhibit positive attitude toward ALHIV	Self-report			
	ALHIV have a positive outlook to life	Self-report			
	Service providers embraced the OTZ model	Self-report			
	Peers Embraced the model and believe it adds value	Self-report			

Note: An item will be awarded a yes/no (Y/N) depending on whether it was done or not.

Rating will be based on a simple 0—not done, 1—partially done, and 2—done as expected. An average score shall be used to quantitatively measure fidelity and categorized as poor (if below 50%, fair if 50–79%, and good if 80 and above).

### Dissemination plan

- Internal dissemination to all TASO staff: TASO center teams and senior management members shall be presented to
- Dissemination to MOH and partners-this will be done virtually
- Manuscript for publication—publication in a peer-reviewed journal, preferably the Journal of International AIDS Society, PLOS ONE or BMJ Open
- National/international conferences: findings from this study shall be presented at both local and international conferences

### Study limitations

- Timeframe maybe inadequate for achieving all the observable outcomes
- Ministry of health definition of HIV VLS may change during the study period, hence affecting study outcomes
- Introduction of another intervention such as the community caregiver model during the course of the study may undermine interpretation of study outcomes.

### COVID-19 and Ebola infection mitigation plan

The project team shall ensure full observance of the Uganda Ministry of Health standard Operating Procedures for infection prevention and control. To this end, hand-washing facilities and use of hand-sanitizers will be implemented throughout the study period. In addition, a blend of both physical and virtual engagements shall be done. Both research teams and participants shall observe physical distance, wear face masks, wash hands, and avoid frequent physical activities as much as possible. Finally, screening using temperature guns will be emphasized at study sites during the same period.

## Discussion

This study aims to adapt and implement the Operation Triple Zero (OTZ) model in TASO Soroti and Mbale COEs in order to achieve both optimal retention and viral load suppression among the ALHIV. The study aims to specifically identify potential barriers and facilitators to both retention and VLS, design effective implementation strategies to adapt the model, and, finally, evaluate its effectiveness in improving treatment outcomes. While the model has been widely used in Kenya and other countries to improve health outcomes among the ALHIV, Uganda is yet to adapt it despite the well documented benefits and the fact that both retention and VLS remain sub-optimal in the country.

Findings from this study are likely to underpin scale up of the model country-wide. The design has been made deliberately elaborate to strategically place both the ALHIV and their caregivers at the center of the intervention. The use of qualitative design is to help understand the perspectives of key stakeholders including the direct beneficiaries themselves and their caregivers who are often left out of intervention designs. In addition, health worker perspectives will be considered to furnish succinct design of implementation strategies. The interactions of both barriers and facilitators will enable strategic and careful kneading of the intervention design in order to maximize outcomes. Importantly, both the strategies and the intervention will be tested to establish their effectiveness in achieving optimal HIV treatment outcomes among the ALHIV. Given the importance of context in implementation science, the lessons learnt in the implementation of OTZ in the two settings will provide the necessary ingredients for other facilities to effectively adapt the intervention in Uganda.

## Data Availability

Data for this study will be made publicly accessible as and when they become available.

## Abbreviations

ART: Antiretroviral therapy: combination of ARVs used in the treatment of HIV
CALHIV: Children and adolescents living with HIV: individuals aged below 20 years who are HIV positive
HIV: Human immunodeficiency virus
AIDS: Acquired immunodeficiency syndrome
TASO: The AIDS Support Organization
MOH: Ministry of Health
OTZ: Operation Triple Zero
RE-AIM: Reach, Effectiveness, Adoption, Implementation and Maintenance: A framework of implementation science used to evaluate interventions in public health services.
VL: HIV viral load
YAPS: Youth and Adolescent Peer Supporter
CFIR: Consolidated Framework for Implementation Research
IAC: Intensive adherence counseling
